# Supplementary material for: Cell surface-bound La protein regulates the cell fusion stage of osteoclastogenesis
Source: Nat Commun. 2023 Feb 4;14:616. doi: 10.1038/s41467-023-36168-x (PMC9899215; doi:10.1038/s41467-023-36168-x)
Supplement: Supplementary file 1 — Supplementary Information [file 41467_2023_36168_MOESM1_ESM.pdf]

Supplementary Materials for

CELL SURFACE-BOUND LA PROTEIN REGULATES THE CELL FUSION STAGE OF OSTEOCLASTOGENESIS

Jarred M. Whitlock[1], Evgenia Leikina[1], Kamran Melikov[1], Luis Fernandez De Castro[2], Sandy Mattijssen[3], Richard J. Maraia[3], Michael T. Collins[2], Leonid V. Chernomordik[1#*]

Correspondence to: jarred.whitlock@nih.gov & chernoml@mail.nih.gov

Supplementary Materials file includes:

Figs. S1 to S7
Supplementary Dataset 1
Source Data
Editorial Policy Checklist
NCOMMS-21-47862C_Author_checklist_1670429707_45
Reporting Summary

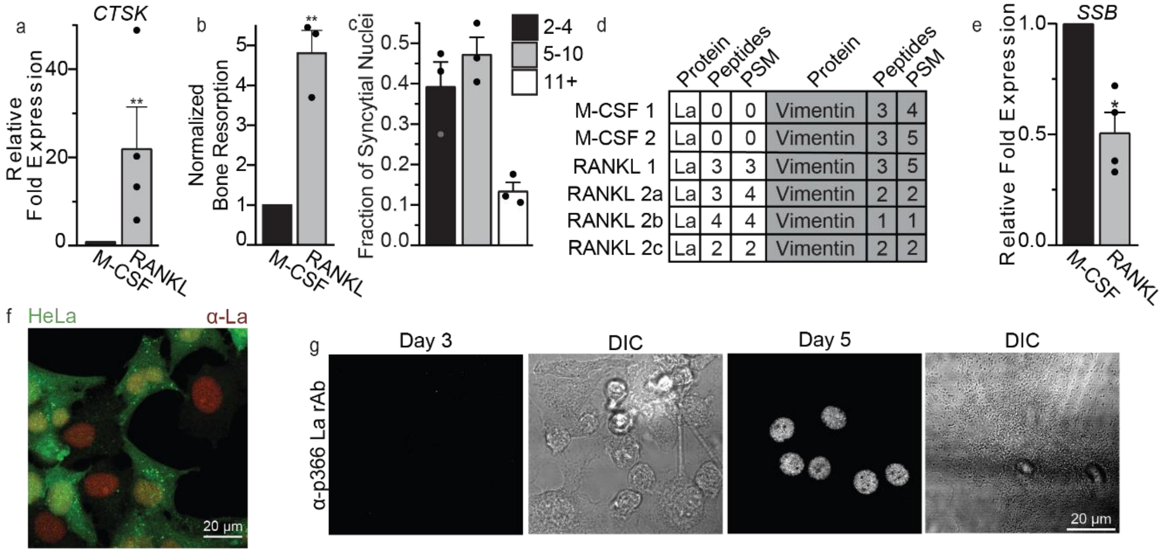

Figure S1: Characterization of monocyte derived osteoclasts and identification of La protein in osteoclasts. (a) qPCR evaluation of the osteoclast differentiation marker *CTSK* from the conditions depicted in Figure 1a. (n=4) (P=0.008) **(b)** Quantification of fluorescent bone resorption in osteoclast precursors (M-CSF) vs differentiated osteoclasts (RANKL). (n=3) (P=0.006) (Day 5 post-RANKL) **(c)** Fraction of nuclei in syncytial osteoclasts of varying sizes. (n=4) (Day 5 post-RANKL) **(d)** Mass spectrometry data from six excised bands separated in six separate lanes ran on a single gel, as seen in Figure 1c (arrowhead indicates band cut). Each lane represented a distinct cell lysate. Cells were collected from two healthy donors and differentiated for six days with M-CSF and further differentiated for three days with M-CSF or M-CSF + RANKL. Vimentin was also detected in each sample, as it has a similar molecular weight to La, but, in contrast to La, vimentin levels were roughly equivalent in M-CSF vs RANKL samples. The table shows the total number of distinct peptide sequences identified in the protein group (Peptides) and the peptide spectrum matches (PSM). Data represent two biological replicates (1&2) and three technical replicates (2a-c). **(e)** qPCR evaluation of SSB (La gene) from the osteoclastogenic stages depicted in **a** and **b** (n=3) (P=0.03). **(f)** Representative immunofluorescence image of α-La mAb staining in GFP expressing HeLa cells. **(g)** Representative immunofluorescence images of α-p366 La rAb staining in primary human osteoclasts during active fusion (Day 3) and when fusion has plateaued (Day 5). (**a, b, e**) Statistical significance was evaluated via two-tailed paired t-test. *= P<0.05; ** = P<0.01. Data are presented as mean values +/- SEM. Source data are provided as a Source Data file.

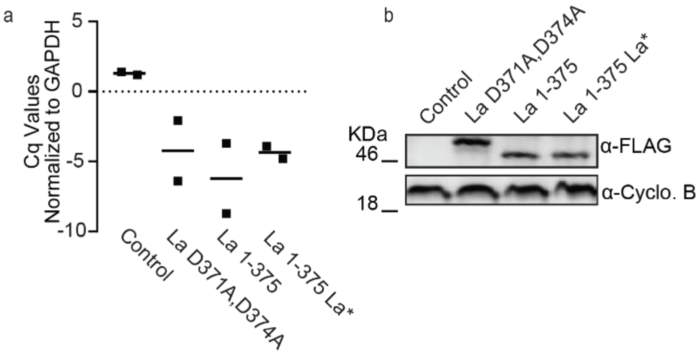

Figure S2: Exogenous La constructs express at similar levels. (a) ΔCq values of La signal in human osteoclasts transfected with empty, La D371A,D374A, La 1-375 and “RNAΔ” La 1-375 Q20A_Y24A_D33I mammalian expression plasmids. GAPDH was used as a housekeeping transcript control. (n=2) **(b)** Representative Western demonstrating the steady-state levels of the FLAG-La constructs used in Figure 2f-i in transfected HeLa cells. Unfortunately, osteoclasts exhibited significant, non-specific FLAG signal producing several bands at 40-60 KDa in lysates from untransfected cells, hindering similar analysis of the changes in La expression following transfection of osteoclasts.

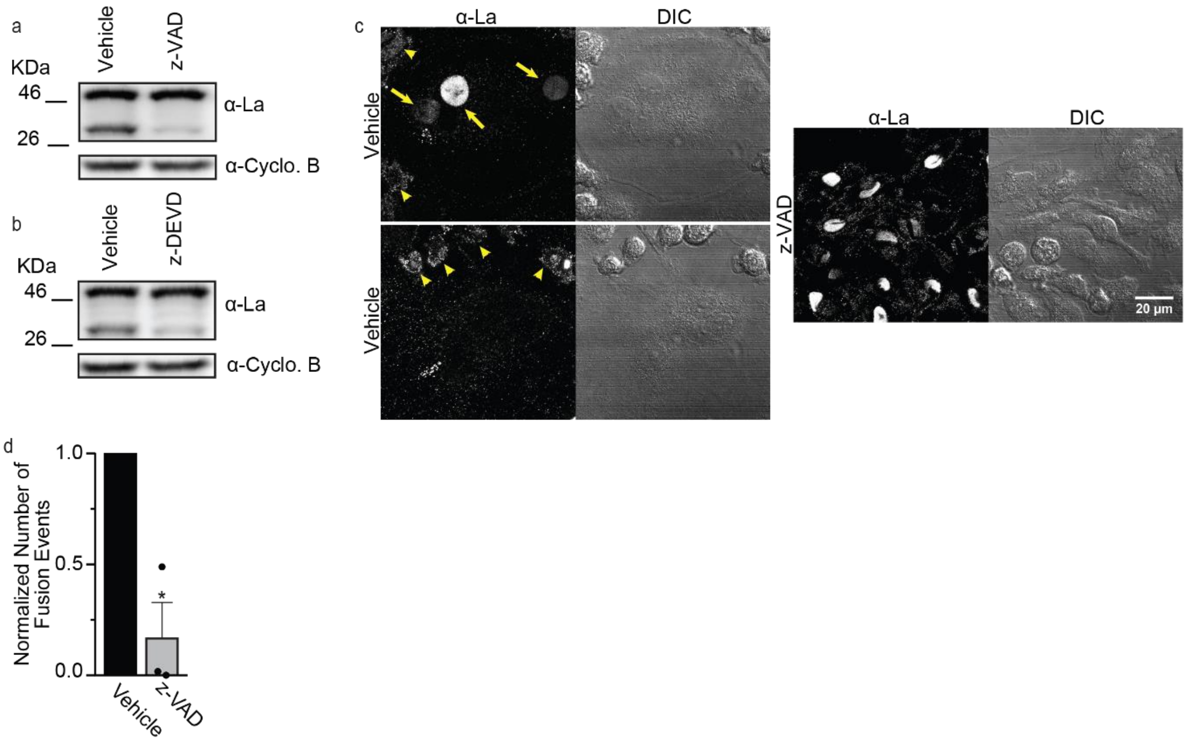

Figure S3: Formation of multinucleated osteoclasts depends on La cleavage. Representative Western blots demonstrating that the pan-caspase inhibitor z-VAD-fmk (**a**) and caspase 3 inhibitor z-DEVD-fmk (**b**) block the production of cleaved La in human osteoclasts (Day 3-4 post-RANKL). **(c)** Representative immunofluorescence images of α-La mAb staining in primary human osteoclasts during active fusion (Day 3) under control conditions (vehicle) and following inhibition of La cleavage via application of z-VAD-fmk. Two images under control conditions are provided to highlight the partial nuclear staining observed in fused cells (top, arrows) and the predominately cytoplasmic staining observed in non-fused cells (bottom, arrowheads) and some fused cells at this timepoint. **(d)** Quantification of the number of fusion events in cells with 3+ nuclei from **c**. (n=3) (P=0.05). Statistical significance was evaluated via one-tailed paired t test. * = P<0.05. (**d**) Data are presented as mean values +/- SEM. Source data are provided as a Source Data file.

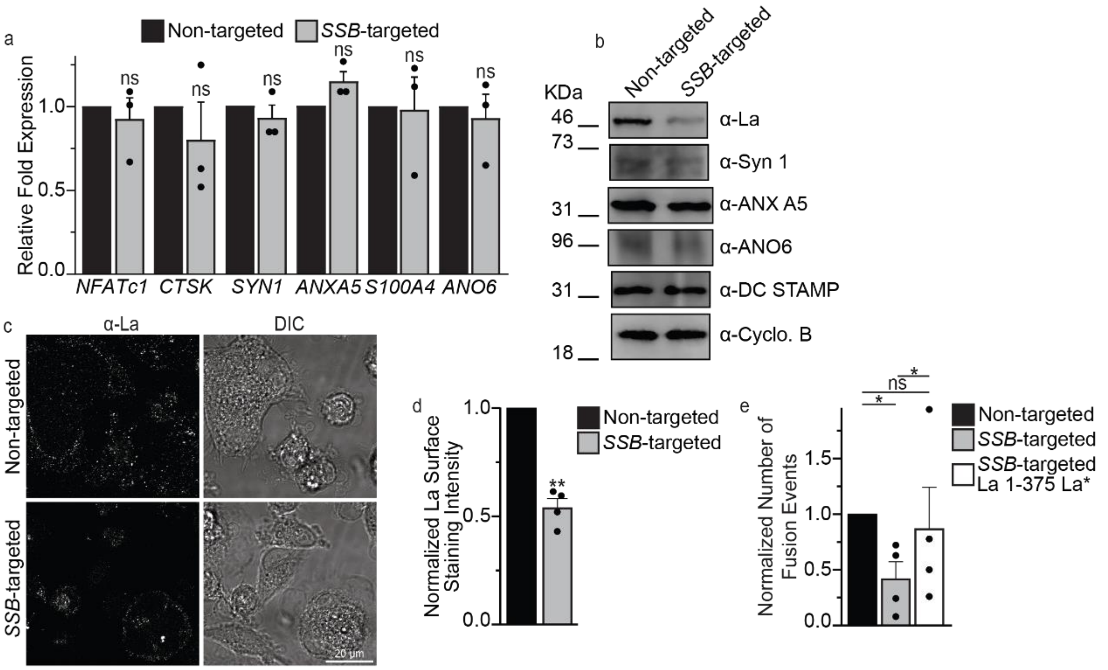

Figure S4: RNAi suppression of La does not alter the steady-state levels of transcripts or proteins implicated in osteoclastogenic differentiation or osteoclast fusion. (**a**) qPCR evaluation of two osteoclast differentiation markers, *NFATc1* and *CTSK* and the osteoclast fusion related transcripts *SYN1*, *ANXA5*, *S100A4* and *ANO6* (TMEM16F) following the siRNA treatments described in Figure 2c. (n=4) (P=0.4, 0.5, 0.5, 0.6, 0.8 and 0.1). (**b**) Representative Western blot demonstrating the specific reduction of La following *SSB*-targeted siRNA treatments compared to osteoclast fusion related proteins SYN1, ANX A5, ANO6 and DC-STAMP vs Cyclo. B loading control. (α-La rAb) (**c**) Representative immunofluorescence images of α-La mAb surface staining in primary human osteoclasts following treatment with non-targeted vs *SSB*-targeted siRNA. (**d**) Quantitative evaluation of α-La mAb surface staining intensity in osteoclasts without membrane permeabilization. (n=4) (P=0.002) (**e**) Quantification of the number of fusion events in RANKL differentiated osteoclasts treated with non-targeted or *SSB*-targeted siRNA with or without addition of La* (La 1-375 Q20A_Y24A_D33I)**.** (n=4) (P= 0.02, 0.3 and 0.02, respectfully). Statistical significance was assessed via two-tailed paired t-test (**a**) and one-tailed paired t-tests (**d** and **e**). *= P<0.05; ** = P<0.01. (**a, d, e**) Data are presented as mean values +/- SEM. Source data are provided as a Source Data file.

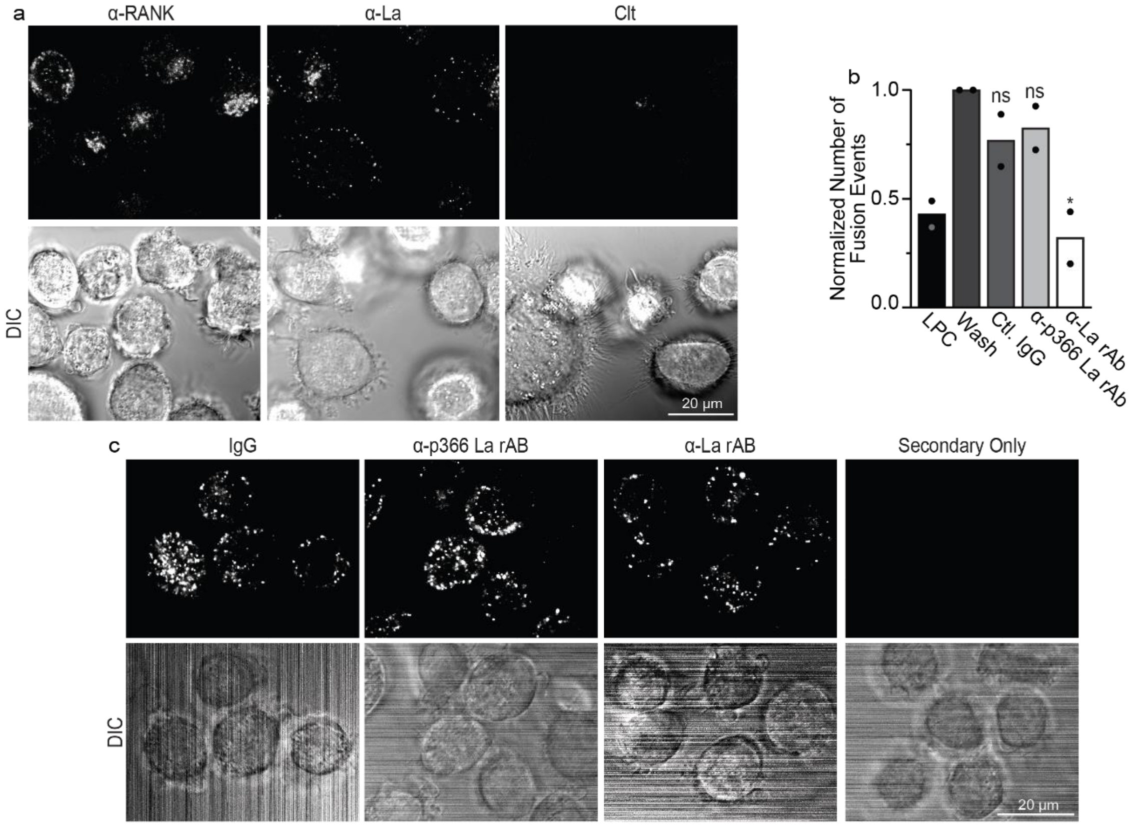

Figure S5: **α-La treatment suppresses synchronized osteoclast membrane fusion**. **(a)** Representative immunofluorescence images of α-RANK vs α-La mAb surface staining in human osteoclasts 10 minutes after LPC wash out. **(b)** The effects of control IgG, α-p366 La rAb or α-La rAb (5µg/ml) application on synchronized fusion of human osteoclasts. Data normalized to those after application of isotype control (rabbit IgG). (n=2) (P=0.08, 0.1 and 0.04, respectively). LPC – fusion observed without removal of LPC. **(c)** Representative immunofluorescence images of IgG, α-p366 La rAb vs α-La rAb surface staining in primary human osteoclasts compared to the cells stained with secondary antibodies alone 10 minutes after LPC wash out, as in **b**. Statistical significance was assessed via one-tailed paired t-test. * = $P<0.05$. Data are presented as mean values +/- SEM. Source data are provided as a Source Data file.

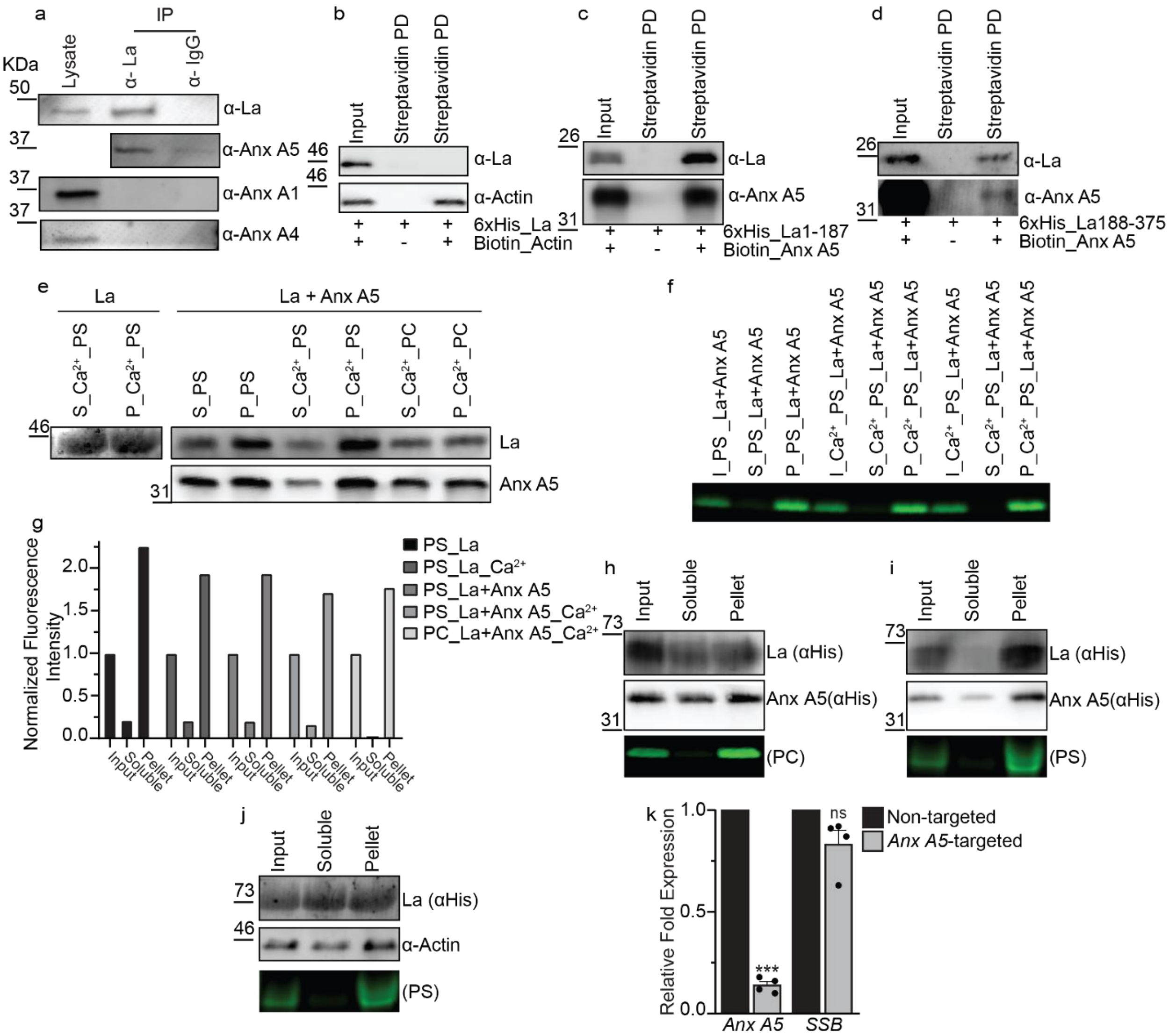

Figure S6: La interacts with Anx A5. (**a**) Immunoprecipitation of osteoclast lysates. La supermolecular complexes were captured on immunomagnetic beads via monoclonal α-La mAb and complexes were blotted using α-La rAb and rabbit antibodies raised towards other targets of interest. (**b**-**d**) Representative Western blots of magnetic, streptavidin pull-downs. (**b**) Biotin-Actin. Lane 1= La+Actin input, Lane 2= La alone pull-down and Lane 3= La+Actin pull-down. (**c**) Biotin-Anx A5. Lane 1= La 1-187+Anx A5 input, Lane 2= La 1-187 alone pull-down and Lane 3= La 1-187+Anx A5 pull-down. (**d**) Biotin-Anx A5. Lane 1= La 188-375+Anx A5 input, Lane 2= La 188-375 alone pull-down and Lane 3= La 188-375+Anx A5 pull-down. (**e**) Western blot assessing soluble vs liposome-enriched fractions presented in Figure 5d. (**f**) Representative image of lissamine rhodamine phosphatidylethanolamine containing bands just before the dye front from the same samples as b demonstrating the effective pelleting of liposomes under conditions used in Figure 5d. (**g**) Densitometry quantification of the lissamine rhodamine phosphatidylethanolamine band intensity from **f**, including lanes for experiments with liposomes incubated with La or La with Ca^{2+}. (**h-j**) Western blot and lissamine rhodamine phosphatidylethanolamine imaging, as in **e** and **f**, for PC liposomes incubated with La and Anx A5 (**h**), for PS containing liposomes incubated with La and Anx A5 (**i**) and for PS containing liposomes incubated with La and Actin (**j**). (**k**) qPCR evaluation of *Anx A5* and *SSB* (La gene) from osteoclast treated with non-targeted or *Anx A5*-targeted siRNA as in Figure 5**e** and **f** (n=4) (P=0.0007 and 0.1, respectively). Note in **e**, **h-i** that 6xhis tagged La 1-408, Anx A5 and Actin run at slightly larger molecular weight because these samples were not heated during sample denaturing. In **f,h-j**, the fluorescent lipid ran with dye front, below m.w. standards. Statistical significance was evaluated

via two-tailed paired t-test. *** = P<0.0001. Data are presented as mean values +/- SEM. Source data are provided as a Source Data file.

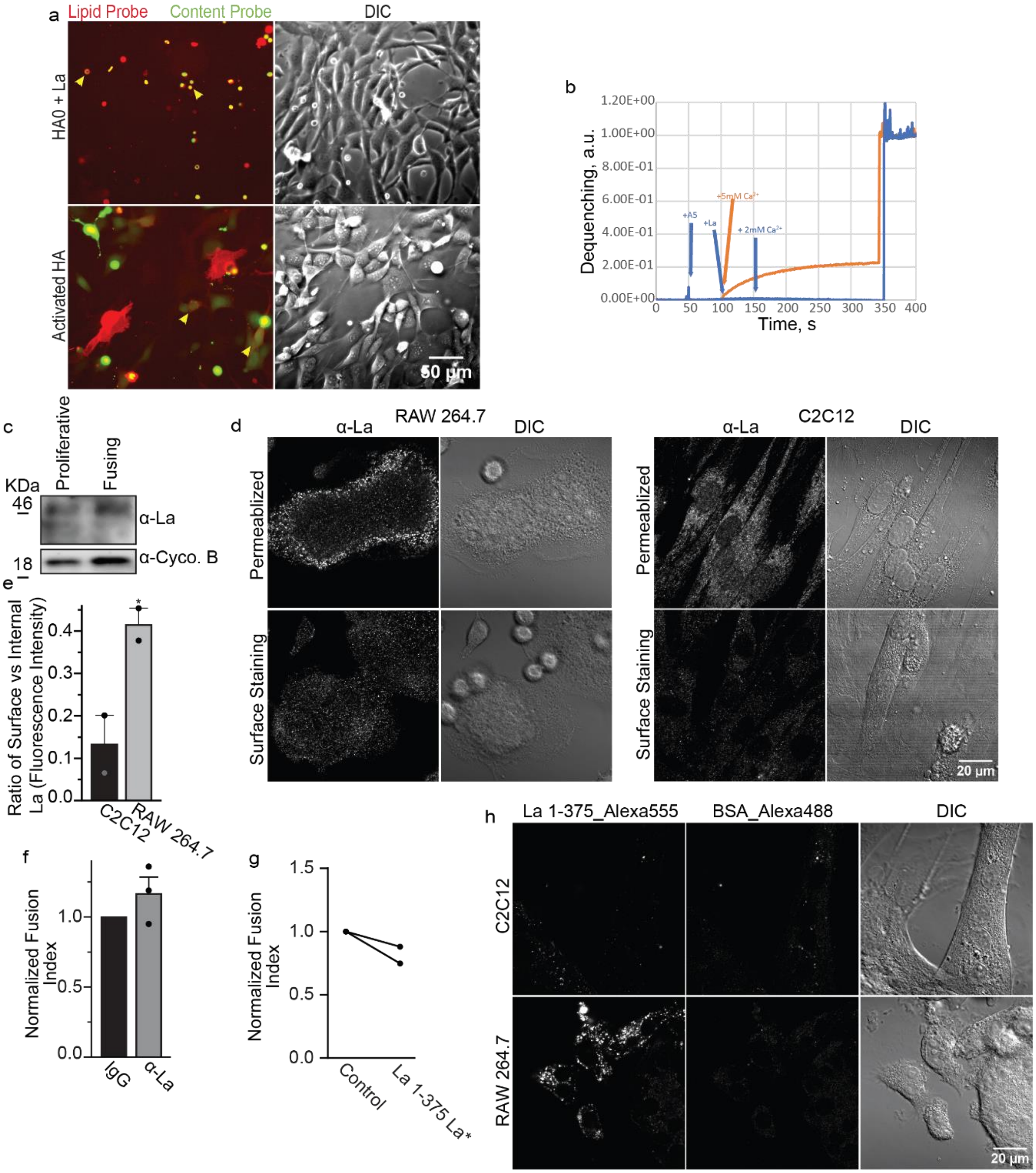

Figure S7: La does not facilitate fusion in either heterologous fusion systems or myoblasts. (**a**) Representative images of HA0 expressing 3T3 fibroblasts bound to RBCs labeled with the fluorescent lipid PKH26 (red) and carboxyfluorescein (green). Fusion was assessed via transfer of content and/or lipid probes after cells were treated with 40nM recombinant La (top) or subjected to tryptic activation and pH 5.0 induced restructuring of HA. Yellow arrowheads denote examples of bound RBCs (top) or labeled

fibroblasts following fusion (bottom). (**b**) Assessment of fluorescent dequenching as a measure of lipid mixing between liposomes upon addition of 13.5 nM Anx A5, 13.5 nM La and 2mM Ca^{2+} or only 5mM Ca^{2+} (positive control). (n=2) (**c**) Representative Western blot of protein lysates from C2C12 myoblasts before and after 3 days differentiation with α-La mAb. (**d**) Representative images of α-La mAb immunofluorescence staining of permeabilized (cell-associated) or non-permeabilized (cell surface) fusing murine osteoclasts (RAW 264.7) vs myoblasts (C2C12) with α-La mAb. (**e**) Quantified ratio of fluorescence intensities of surface La vs cell-associated La, in images such as in **d**. (n=2) (P=0.03) (**f**) Quantification of fusion index (nuclei in syncytia divided by total number of nuclei) in differentiated C2C12 myoblasts treated with 5μg/ml isotype control IgG vs α-La mAb. Data normalized to IgG. (n=3) (P=0.2). (**g**) Quantification of fusion index in differentiated C2C12 myoblasts in control conditions or when 40nM La 1-375 La* was added, normalized to control. (n=2) (P=0.09) (**h**) Representative fluorescence imaging of fusing C2C12 myoblasts or RAW 264.7 osteoclasts in the presence of fluorescently tagged La 1-375 and BSA. (**e, f, g**) Statistical significance was assessed via one-tailed paired t-test. * = P<0.05. Data are presented as mean values +/- SEM. Source data are provided as a Source Data file.

.
