# Supplementary material for: Cell surface-bound La protein regulates the cell fusion stage of osteoclastogenesis
Source: Nat Commun. 2023 Feb 4;14:616. doi: 10.1038/s41467-023-36168-x (PMC9899215; doi:10.1038/s41467-023-36168-x)
Supplement: Supplementary file 4 — Reporting Summary [file 41467_2023_36168_MOESM4_ESM.pdf]

nature portfolio

Corresponding author(s): Jarred Whitlock and Leonid Chernomordik

Last updated by author(s): Dec 28, 2022

Reporting Summary

Nature Portfolio wishes to improve the reproducibility of the work that we publish. This form provides structure for consistency and transparency in reporting. For further information on Nature Portfolio policies, see our Editorial Policies and the Editorial Policy Checklist.

Statistics

For all statistical analyses, confirm that the following items are present in the figure legend, table legend, main text, or Methods section.

n/a	Confirmed	
☐	☒	The exact sample size (*n*) for each experimental group/condition, given as a discrete number and unit of measurement
☐	☒	A statement on whether measurements were taken from distinct samples or whether the same sample was measured repeatedly
☐	☒	The statistical test(s) used AND whether they are one- or two-sided<br>*Only common tests should be described solely by name; describe more complex techniques in the Methods section.*
☒	☐	A description of all covariates tested
☐	☒	A description of any assumptions or corrections, such as tests of normality and adjustment for multiple comparisons
☐	☒	A full description of the statistical parameters including central tendency (e.g. means) or other basic estimates (e.g. regression coefficient) AND variation (e.g. standard deviation) or associated estimates of uncertainty (e.g. confidence intervals)
☐	☒	For null hypothesis testing, the test statistic (e.g. *F*, *t*, *r*) with confidence intervals, effect sizes, degrees of freedom and *P* value noted<br>*Give P values as exact values whenever suitable.*
☒	☐	For Bayesian analysis, information on the choice of priors and Markov chain Monte Carlo settings
☒	☐	For hierarchical and complex designs, identification of the appropriate level for tests and full reporting of outcomes
☒	☐	Estimates of effect sizes (e.g. Cohen's *d*, Pearson's *r*), indicating how they were calculated

Our web collection on statistics for biologists contains articles on many of the points above.

Software and code

Policy information about availability of computer code

Data collection	FluoroEssence - version 2.1;<br>MicroManager – version 1.4.23;<br>Fiji – version 2.1.0/1.53c;<br>RStudio – version 1.2.<br>Fiji/ImageJ open-source image processing package v.2.1.0/1.53c<br>Proteome Discoverer software v1.4
Data analysis	Fiji image processing package for viewing and scoring and Graphpad Prism- version 8 for statistical analysis.

For manuscripts utilizing custom algorithms or software that are central to the research but not yet described in published literature, software must be made available to editors and reviewers. We strongly encourage code deposition in a community repository (e.g. GitHub). See the Nature Portfolio guidelines for submitting code & software for further information.

Data

Policy information about availability of data

All manuscripts must include a data availability statement. This statement should provide the following information, where applicable:

- Accession codes, unique identifiers, or web links for publicly available datasets
- A description of any restrictions on data availability
- For clinical datasets or third party data, please ensure that the statement adheres to our policy

All source data are provided with this paper.

Human research participants

Policy information about studies involving human research participants and Sex and Gender in Research.

Reporting on sex and gender	n.a.
Population characteristics	n.a.
Recruitment	n.a.
Ethics oversight	Anonymized human cells were obtained with approval by Institutional Review Boards and with informed consent. Elutriated monocytes from healthy donors were obtained through the Department of Transfusion Medicine at National Institutes of Health under protocol 99-CC-0168 approved by the National Institutes of Health Institutional Review Board. Research blood donors provided written informed consent and blood samples were de-identified prior to distribution, Clinical Trials Number: NCT00001846. We also used elutriated monocytes from healthy donors obtained through Elutriation Core Facility, University of Nebraska Medical Center, informed consent was obtained under an Institutional Review Board approved protocol for human subject research 0417-22- FB. Research blood donors provided informed consent and samples were de-identified prior to distribution.

Note that full information on the approval of the study protocol must also be provided in the manuscript.

Field-specific reporting

Please select the one below that is the best fit for your research. If you are not sure, read the appropriate sections before making your selection.

☒ Life sciences ☐ Behavioural & social sciences ☐ Ecological, evolutionary & environmental sciences

For a reference copy of the document with all sections, see nature.com/documents/nr-reporting-summary-flat.pdf

Life sciences study design

All studies must disclose on these points even when the disclosure is negative.

Sample size	No sample size calculation was performed. Samples sizes that we chose throughout our manuscript, including at least 8 fields of view per condition in experiment, were based on preliminary experiments and were considered sufficient as long as the differences/or the lack of differences were statistically significant and reproducible in independent experiments. All n values are clearly indicated within the figure and figure legends.
Data exclusions	No data was excluded from the analysis
Replication	If not stated otherwise, all experiments were performed at least three times with similar results.
Randomization	The samples and cell culture dishes used for controls and experiments were allocated randomly and all image fields were randomly selected.
Blinding	The investigators were not blinded to group allocation. Our data analysis is based on the objectively measurable data: percentage of nuclei in syncytia and numbers of labeled cells.

Reporting for specific materials, systems and methods

We require information from authors about some types of materials, experimental systems and methods used in many studies. Here, indicate whether each material, system or method listed is relevant to your study. If you are not sure if a list item applies to your research, read the appropriate section before selecting a response.

Materials & experimental systems

n/a	Involved in the study
☐	☒ Antibodies
☐	☒ Eukaryotic cell lines
☒	☐ Palaeontology and archaeology
☐	☒ Animals and other organisms
☒	☐ Clinical data
☒	☐ Dual use research of concern

Methods

n/a	Involved in the study
☒	☐ ChIP-seq
☒	☐ Flow cytometry
☒	☐ MRI-based neuroimaging

Antibodies

Antibodies used

anti-Cyclophilin B (Cell Signaling Technology, D1VdJ Rabbit mAb #43603), anti-GAPDH (CST, D16H11), anti-Tubulin (Abcam, 7750), anti-RANK (Abcam, 13918), anti-Anx A5 (Abcam, 54775), control rabbit polyclonal IgG (Abcam, 27478), IgG2a (Abcam, 18415); anti-La, Abcam, 75927), IgG1 (Abcam, 170190); anti--Anx A5, Abcam, 54775), α-Anx A1 (Abcam, 47661), α-Anx A4 (Abcam, 65846), α-6xHis (Abcam, 18184) and-FISH (Abcam, 118575). α -La mAb; Abcam, 75927); rabbit Anti-SSB antibody (Invitrogen, PA5-29763); rabbit anti-La Phospho-Ser366 antibody (Abcam, 61800); Anti-mouse IgG Fab2 Alexa Flour 488 (Cell Signaling Technology, Catalogue # 4408S) Anti-rabbit IgG (H+L), F(ab')2 Fragment (Alexa Fluor® 647 Conjugate) #4414S

Validation

α -La antibody (α -La mAb; Abcam, 75927) has been validated by the following findings: (1) a drastic increase in La content between monocytes treated with M-CSF and RANKL vs. those treated only with M-CSF detected by mass spectroscopy analysis results in drastic increase in La bands in Westerns and in La staining in immunofluorescence detected with this Ab (Fig. S1 and Fig. 1). (2) siRNA suppression of La expression lowers La staining with this mAb (Fig. 2, Fig. S4d). (3) the presence of La in protein complexes isolated by immunoprecipitation with this mAb has been confirmed with another α-La Ab (rabbit Anti-SSB antibody Invitrogen, PA5-29763, referred to as α-La rAb) (Fig. S6a). In addition to α-La mAb and α -La rAb, in some experiments we also used rabbit anti-La Phospho-Ser366 antibody (Abcam, 61800, referred to as α-p366 La rAb) that specifically recognizes phosphorylated human La (phosphoSer366). The specificities of La recognition in immunofluorescence microscopy for α-La rAb and α-p366 La rAb were mutually validated by showing the same nuclear staining at the late stage of osteoclast formation (5 days after RANKL application), when phosphorylated La returns to nuclei 33. The specificity of α-La rAb in Westerns has been confirmed by the experiments, in which we suppressed La expression with siRNA (Fig. S4b).

Antibodies α-Cyclophilin B (Cell Signaling Technology, D1VdJ Rabbit mAb #43603), α-GAPDH (Cell Signaling Technology, D16H11), α-Tubulin (Abcam, 7750), α-RANK (Abcam, 13918), and α-Anx A5 (Abcam, 54775) were all validated by the manufacturers.

Eukaryotic cell lines

Policy information about cell lines and Sex and Gender in Research

Cell line source(s)

Raw 264.7 and C2C12 cells (ATCC);
HA-expressing NIH 3T3 mouse fibroblasts of clone 15 cell line (J. Zimmerberg's lab, Wilson, R. L. et al. Hemagglutinin clusters in the plasma membrane are not enriched with cholesterol and sphingolipids. Biophys J 108, 1652-1659, doi:10.1016/j.bpj.2015.02.026 (2015).);
Human Red Blood Cells, Research Donor Program (Bethesda, MD)

Authentication

None of these cell lines were authenticated beyond the standard authentication carried out for all lines purchased from ATCC.

Mycoplasma contamination

All cell lines are routinely tested negative for mycoplasma contamination.

Commonly misidentified lines
(See ICLAC register)

We have not used any commonly misidentified lines listed in the ICLAC Table.

Animals and other research organisms

Policy information about studies involving animals; ARRIVE guidelines recommended for reporting animal research, and Sex and Gender in Research

Laboratory animals

A mouse model of fibrous dysplasia with inducible expression of hyperactive GαsR201C in cells of the osteogenic lineage 56 was used to obtain bone marrow explants (described below). For this study we used 12–18-week-old females generated by genetic cross of C57BL/6 and FVB/N strains. Mice were separated by sex at weaning and housed in shared cages of maximum 5 littermates in a conventional veterinary facility (with quarantine requirements, and exclusion of specific pathogens) with a 12 h/12 h light-dark cycle and fed ad libitum with NIH 07 (autoclavable) hard diet (Envigo, Frederick, MD).

Wild animals

no wild animals were used in the study

Reporting on sex

females

Field-collected samples

No field collected samples were used in the study.

Ethics oversight	NIH-Intramural Animal Care and Use Committee (ACUC) of the National Institute of Dental and Craniofacial Research approved protocols (ASP #19-897), in compliance with the Guide for the Care and Use of Laboratory Animals.

Note that full information on the approval of the study protocol must also be provided in the manuscript.
